# Comparison of the In Vitro Mutagenicity of Artemisia draconculus L. With Sodium Dichromate by Performing Single Cell Gel Electrophoresis (SCGE) or the Comet Assay

**DOI:** 10.17795/jjnpp-3561

**Published:** 2012-01-04

**Authors:** Heibatullah Kalantari, Hamid Galehdari, Zahra Zaree, Hadi Kalantar, Golnaz Varnasary

**Affiliations:** 1 School of Pharmacy, Toxicology Research Center, Jundishapur University of Medical Sciences, Ahvaz, IR Iran

**Keywords:** Comet Assay, Mutagenicity, *Artemisia*

## Abstract

**Background:**

The increasing use of herbal drugs and their easy availability have necessitated the use of mutagenicity tests to analyze their toxicity and safety.

**Objectives:**

The aim of this study was to determine the in vitro mutagenicity of Artemisia draconculus L., a herbal drug, by performing single cell gel electrophoresis (SCGE).

**Materials and Methods:**

In this study, we obtained a herbal drug with A. draconculus at a density of 0.94; doses of 100 μl, 200 μl, 400 μl, and 800 μl equivalent to 94 mg, 188 mg, 376 mg, and 752 mg of A. draconculus, respectively, were used. Sodium dichromate at a dose of 262 mg was considered to be the positive control, and blood was considered to be the negative control. Blood samples were centrifuged at 3500 rpm for 5 min, and the lower portion of the residue was isolated and mixed with low melting point agarose.

**Results:**

A cell suspension was prepared and applied on pre-coated agarose gel slides. Lysis, electrophoresis under alkaline conditions, staining of DNA, comet visualization, and comet scoring were carried out. The statistical analysis of the obtained results showed that with an increase in the dosage of A. draconculus, DNA damage also increased significantly (P < 0.05).

**Conclusions:**

These findings provide valuable information regarding the safety and toxicity of this herbal drug, and this information will be helpful in ensuring rational use of this drug.

## 1. Background

The increasing popularity of over-the-counter medicinal products and other natural drugs have had a significant effect on the health care system. A wide variety of herbal products are easily accessible and commercially available in various forms and dosages; to ensure appropriate use of these drugs, however, it is necessary to perform mutagenicity tests and establish their toxicity. Hence, in this study, we aimed to analyze the mutagenicity of the herbal drug A. draconculus. A large number of tests such as the micronucleus test and the single cell gel electrophoresis (SCGE) or comet assay are available for screening and evaluation of the safety of such products (1, 2).

A. draconculus, an aromatic herb of the Compositae family, is widely used as a mild sedative and in the treatment of toothache. A. draconulus also promotes the production of bile in the liver, which aids digestion and helps accelerate the process of elimination of toxic waste from the body (3). Over the past decade, SCGE has become the standard method to assess DNA damage, and it is applied in genotoxicity testing, human biomonitoring, molecular epidemiology, and in fundamental research on DNA damage and repair. The assay is simple, sensitive, versatile, quick, and economical. In 1970, Peter Cook and his colleagues developed an approach to investigate the nuclear structure of cells lysed using a non-ionic detergent. This technique was further developed by Swedish researches Ostiling and Johanson in 1984 and modified by N.P. Singh et al in 1988, after which it came to be known as the alkaline comet assay (4–6).

## 2. Objectives

The aim of this study was to determine the in vitro mutagenicity of Artemisia draconculus L., a herbal drug, by performing single cell gel electrophoresis (SCGE).

## 3. Materials and Methods

Reagents of the highest purity grades and materials such as agarose low melting point were purchased from Fluka Japan. Dimethyl sulfoxide (DMSO), sodium dichromate, ethidium bromide, and Triton X-100 were purchased from Merck (Germany). Disodium ethylenediaminetetraaceticacid, N-laury sarcosin sodium salt, Trizma (tris) were purchased from Sigma. The herbal drug (Tarkhon) was purchased from Barich herbal drug Co. Iran. Electrophoresis apparatus was obtained from Shandon Vokam (England); the centrifuge was from Backman (TJ-6; USA); and the fluorescence microscope (H600 AFL50/100) was from Helmat Hund (Germany). Hanks’ balanced salt solution (HBBS), phosphate-buffered saline (PBS), lysis solution, neutral buffer, and the staining solution were prepared according to the Mckelvey-Martin procedure with minor modifications (7). All other working solutions which were required for this experiment were obtained from our toxicology laboratory. We determined the density of A. draconculus in the drug by using a pycnometer, and a density value of 0.94 was calculated using the formula d = m/v. Then dosages of 100 µl, 200 µl, 400 µl, and 800 µl, which were equivalent to 94 mg, 188 mg, 376 mg, and 752 mg of A. draconculus were used in the study. We numbered 2-ml screw-cap tubes and then added 1 ml HBSS, 10 µl blood, and the required amount of A. draconculus (94 mg, 188 mg, 376 mg, and 752 mg) to the screw-cap tubes. Sodium dichromate at a dose of 262 mg was considered as the positive control, and blood was the negative control. We incubated all the screw-cap tubes in a water bath at 37°C, after which the contents of all the tubes were centrifuged for 5 min at 3500 rpm. The upper layer was discarded, and 100 µl of low melting point agarose 0.5% in PBS was added to the lower layer (pellet) in each tube; the tube was then well mixed, the cell suspension was poured on pre-coated agarose microscopic slide, and a cover slip was placed over it until it solidified. After solidification, the cover slip was removed, and all the slides were kept in a lysing solution at 4°C for 1 h. Then the prepared slides were electrophoresed in alkaline buffer for an hour at 25 V and 300 mA. All the slides were then removed from the electrophoresis tank, placed horizontally, and washed gently by flooding them slowly with neutral buffer (3 times) to remove the alkali and detergents, which could interfere with ethidium bromide staining. After 5 min, the slides were stained with ethidium bromide. Lastly, the slides were observed under a fluorescent microscope one by one and analyzed by the Kobayashi method (8-10). The migration pattern was determined, and the comets were estimated according to the following formula: M = (0 NMC + 1 SMC + 2 MMC + 3 LMC)/150

NMC = Cells showing no migration (type 1)

SMC = Cells showing minimal migration (type 2)

MMC = Cells showing intermediate migration (type 3)

LMC = Cells showing long migration (type 4)

## 4. Results

**Figure 1:**
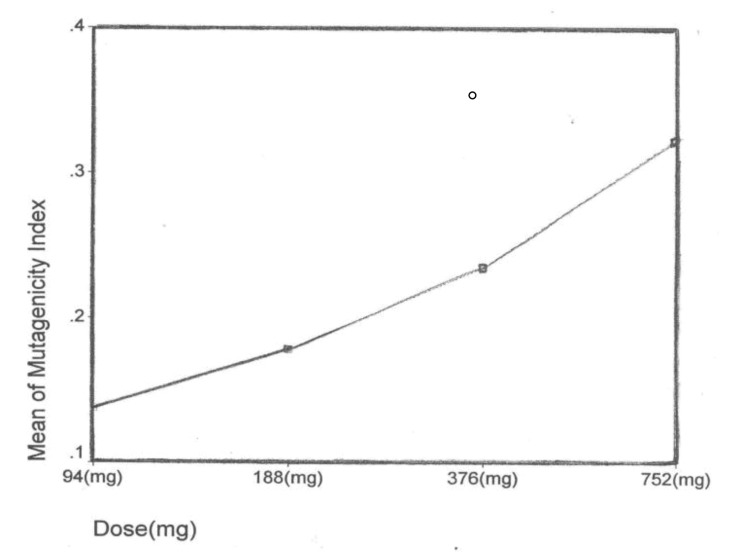
The mutagenicity index of *Artemisia draconculus* of human lymphocytes after exposure to different doses of the drug

**Figure 2:**
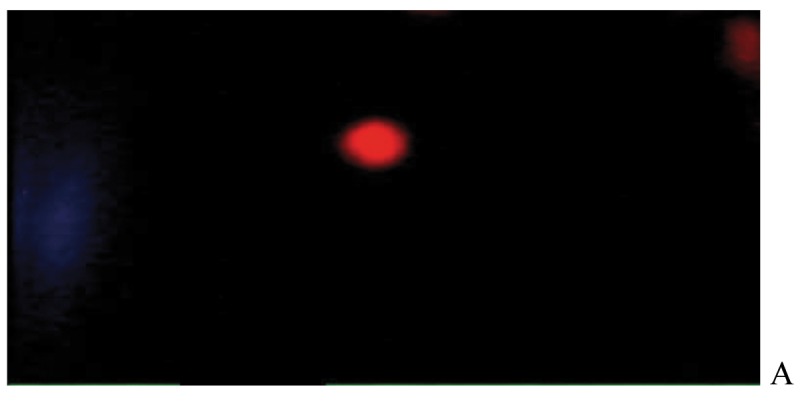
Fluorescence microscopy of human lymphocyte with the negative control

**Figure 3:**
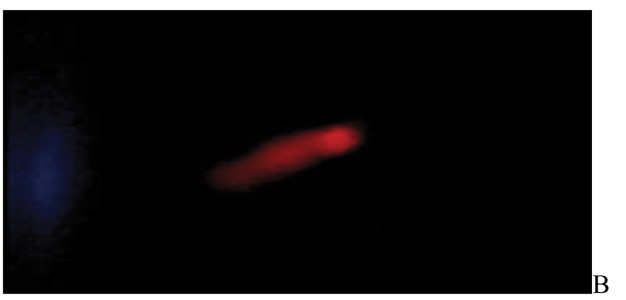
Fluorescence microscopy of human lymphocyte with the positive control

**Figure 4:**
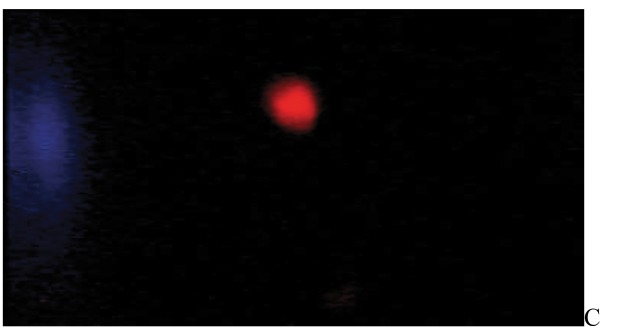
Fluorescence microscopy of human lymphocyte with *Artemisia draconculus* at a dose of 94 gm

**Figure 5:**
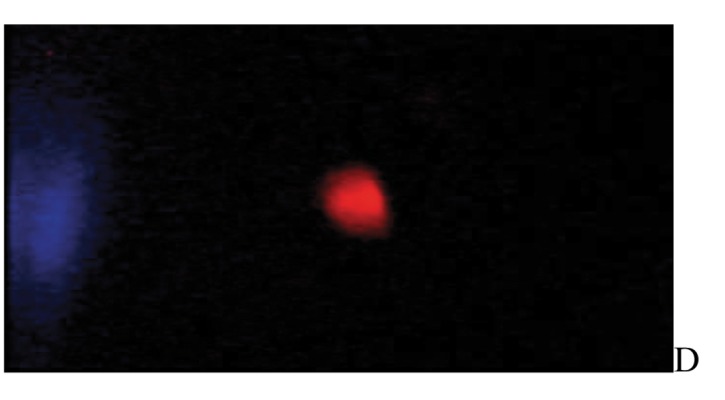
Fluorescence microscopy of human lymphocyte with *Artemisia draconculus* at a dose of 188 gm

**Figure 6:**
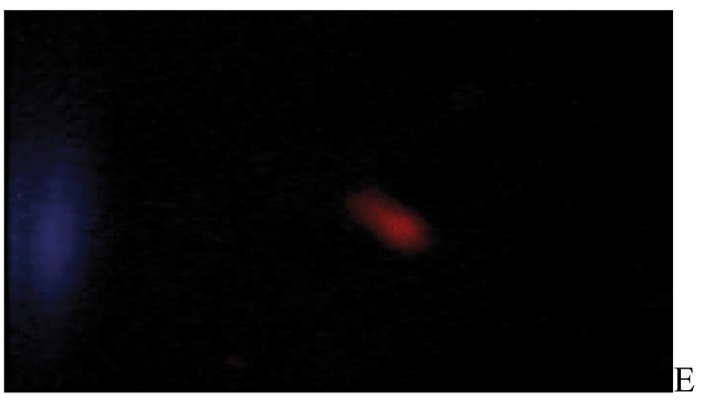
Fluorescence microscopy of human lymphocyte with *Artemisia draconculus* at a dose of 376 gm

**Figure 7:**
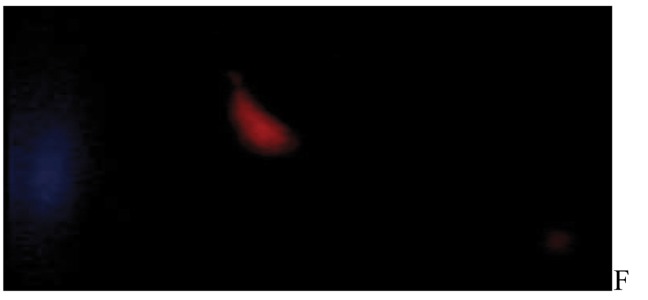
Fluorescence microscopy of human lymphocyte with *Artemisia draconculus* at a dose of 752 gm

After the ethidium bromide staining, fluorescence microscopy showed DNA damage in some cells. The results of different doses of A. draconculus have been summarized in Table 1. Figure 1 indicates the mutagenicity index (MI) of human lymphocytes after exposure to different doses of A. draconculus. As is evident on the curve, points on the curve are the mean values obtained from 4 replicate experiments. We observed comet formations in the different groups microscopically (Figures 2, 3, 4, 5, 6, 7) and compared the comet formation of the drug with that of the positive control (sodium dichromate).

**Table 1:** Effects of different doses of Artemisia draconculus on the DNA of human lymphocyte cells

Groups	*A. draconculus*	*A. draconculus*	*A. draconculus*	*A. draconculus*	Sodium dichromate (positive control)	Buffer (negative control)
Dose	94 g	188 g	376 g	752 g	-	-
Type 1 (no migration)	32	28	24	13	0	39
Type 2 (minimal migration)	16	17	16	21	0	8
Type 3 (intermediate migration)	4	5	7	8	42	3
Type 4 (long migration)	0	0	1	4	8	0
Mutagenicity Index	0.163	0.180	0.235	0.323	0.710	0.092

## 5. Discussion

To assess the potential mutagenic properties of A. draconculus, human lymphocytes were exposed to different doses of the drug. The results of a single test cannot provide a completely accurate assessment of the genotoxicity of a drug. Therefore, it is necessary to employ a variety of genotoxic tests such as micronucleus test, SOS chrome test, and Ames test. Since the knowledge of potential adverse effects of most herbal medicines is limited, identifying the safest and most effective therapeutic dose and promoting their rational use becomes more difficult. Efforts must be made to promote the rational use of the safest and most effective therapies, and in this respect, accurate determination of mutagenicity is crucial. In this study, the genotoxicity of A. draconculus was analyzed by performing SCGE (also known as the comet assay), which is a sensitive and reliable technique for genotoxic evaluation of chemicals and natural pharmaceutical products. The comet assay is based on the intensity of migration of DNA. In a normal DNA, migration occurs in the region of the nucleous, but in damaged DNA, due to the electric charge in electrophoresis, DNA moves away from the nucleous and forms a comet that can be easily observed microscopically. In this study, sodium dichromate, a potent mutagenic agent, was used as the positive control. Four different doses of A. draconculus were compared with sodium dichromate. The relation between MI and the different doses has been described in the form of fluorescence microscopy images, tables, and figures. Statistical analysis of our results showed that DNA damage significantly increased with an increase in the dose of A. draconculus. These finding provide valuable information on the safety and toxicity of this herbal drug, which can be employed to ensure rational use of this drug.

No data on the mutagenicity of A. draconculus has been reported previously. We estimated the mutagenicity of A. draconculus at 4 different dosage levels, and the statistical analysis of the obtained results indicate that with an increase in the dose of A. draconculus, DNA damage also increases significantly. This finding provides valuable data on the safety and toxicity of this herbal drug, which should be considered for a rational use of this drug. Nevertheless, further studies are required to define both the nature and implications of the dose response curve at high levels of exposure to this herbal medicine.
